# Association of Human Leukocyte Antigen with Interstitial Lung Disease in Rheumatoid Arthritis: A Protective Role for Shared Epitope

**DOI:** 10.1371/journal.pone.0033133

**Published:** 2012-05-07

**Authors:** Hiroshi Furukawa, Shomi Oka, Kota Shimada, Shoji Sugii, Jun Ohashi, Toshihiro Matsui, Tatsuoh Ikenaka, Hisanori Nakayama, Atsushi Hashimoto, Hirokazu Takaoka, Yoshiyuki Arinuma, Yuko Okazaki, Hidekazu Futami, Akiko Komiya, Naoshi Fukui, Tadashi Nakamura, Kiyoshi Migita, Akiko Suda, Shouhei Nagaoka, Naoyuki Tsuchiya, Shigeto Tohma

**Affiliations:** 1 Clinical Research Center for Allergy and Rheumatology, Sagamihara Hospital, National Hospital Organization, Sagamihara, Kanagawa, Japan; 2 Department of Rheumatology, Sagamihara Hospital, National Hospital Organization, Sagamihara, Kanagawa, Japan; 3 Tokyo Metropolitan Tama Medical Center, Musashi-dai, Fuchu, Japan; 4 Molecular and Genetic Epidemiology Laboratory, Faculty of Medicine, University of Tsukuba, Tsukuba, Ibaraki, Japan; 5 Kumamoto Center for Arthritis and Rheumatology, Kuhonji, Kumamoto, Japan; 6 Nagasaki Medical Center, National Hospital Organization, Kuhara, Omura, Japan; 7 Department of Rheumatology, Yokohama Minami Kyosai Hospital, Kanazawa-ku, Yokohama, Japan; Albert Einstein Institute for Research and Education, Brazil

## Abstract

**Introduction:**

Interstitial Lung Disease (ILD) is frequently associated with Rheumatoid Arthritis (RA) as one of extra-articular manifestations. Many studies for Human Leukocyte Antigen (HLA) allelic association with RA have been reported, but few have been validated in an RA subpopulation with ILD. In this study, we investigated the association of *HLA class II* alleles with ILD in RA.

**Methods:**

An association study was conducted on *HLA-DRB1, DQB1, and DPB1* in 450 Japanese RA patients that were or were not diagnosed with ILD, based on the findings of computed tomography images of the chest.

**Results:**

Unexpectedly, HLA-DRB1*04 (corrected *P* [*Pc*] = 0.0054, odds ratio [OR] 0.57), shared epitope (SE) (*P* = 0.0055, OR 0.66) and DQB1*04 (*Pc* = 0.0036, OR 0.57) were associated with significantly decreased risk of ILD. In contrast, DRB1*16 (*Pc* = 0.0372, OR 15.21), DR2 serological group (DRB1*15 and *16 alleles) (*P* = 0.0020, OR 1.75) and DQB1*06 (*Pc* = 0.0333, OR 1.57, respectively) were significantly associated with risk of ILD.

**Conclusion:**

*HLA-DRB1* SE was associated with reduced, while DR2 serological group (DRB1*15 and *16) with increased, risk for ILD in Japanese patients with RA.

## Introduction

Rheumatoid arthritis (RA) is a chronic systemic inflammatory disease that affects about 1% of population and is associated with the development of extra-articular manifestations. RA pathogenesis is multifactorial and the disease susceptibility is associated with genetic and environmental factors [1], [2], [3]. Human Leukocyte Antigen (HLA) is known to be associated with RA in most ethnic groups. Some *HLA-DR* alleles are reported to be associated with RA susceptibility [4]. A conserved amino acid sequence at position 70–74 (QKRAA, RRRAA, or QRRAA) in HLA-DRβ chain is shared between the RA-associated *HLA-DR* alleles; this was designated as shared epitope (SE) [4]. Several studies have reported the association of extra-articular manifestations of RA with SE and DR4 subtype [5]. DRB1*04∶01 and *04∶05 were associated with extra-articular manifestations of RA in European and East Asian populations, respectively [6], [7]. Extra-articular manifestations of RA include pericarditis, pleuritis, Felty’s syndrome, vasculitis involving various organs, and interstitial lung disease (ILD). DRB1*04∶01 was strongly associated with Felty’s syndrome in European population and a gene dosage effect was noted [6], [8].

ILD is frequently associated with RA or other collagen-related diseases and named collagen vascular disease-associated ILD (CVD-ILD). CVD-ILD in RA is one of the extra-articular manifestations that influence the prognosis of RA [9]. Recent study reported that median survival following diagnosis of ILD was three years [10]. It is important to elucidate the pathogenesis of ILD complicated with RA. Although association of SE and extra-articular manifestations of RA was reported in several populations, few studies have focused on ILD in RA [11]. In this study, we investigated the association of *HLA class II* with ILD in RA.

**Table 1:** Characteristics of RA patients.

	ILD(+)RA	ILD(−)RA	*P*
Number	129	321	
Mean age, years (SD)	69.5 (7.8)	61.7 (11.2)	*2.3×10^−15^
Male, n (%)	42 (32.6)	47 (14.6)	1.6×10^−5^
Current or past smokers, n (%)	42 (32.6)	88 (27.4)	0.2763
Disease duration, years (SD)	17.1 (12.2)	13.5 (10.1)	*0.0041
Rheumatoid factor positive, n (%)	122 (94.6)	270 (84.1)	0.0027
Anti-citrullinated peptide antibody positive, n (%)	116 (89.9)	278 (86.6)	0.3349
Steinbrocker stage III and IV, n (%)	84 (65.1)	177 (55.1)	0.0525

ILD(+)RA: ILD positive RA, ILD(−)RA: ILD negative RA. Association was tested by chi-square analysis using 2×2 contingency tables or Student’s t-test.

* Student’s t-test was employed.

## Materials and Methods

### Patients and Controls

One hundred twenty nine RA patients with ILD (ILD positive RA group) and 321 without ILD (ILD negative RA group) were recruited at Sagamihara Hospital. Fifty seven healthy controls were recruited at Sagamihara Hospital. In addition, 146 independent RA patients for replication study were recruited at Kumamoto Center for Arthritis and Rheumatology, Nagasaki Medical Center, and Yokohama Minami Kyosai Hospital. These patients did not overlap with previous study participants [11], [12]. All patients and healthy individuals were native Japanese living in Japan. All patients with RA fulfilled the 1988 American College of Rheumatology Criteria for RA [13]. This study was reviewed and approved by the research ethics committees of each participating institute, Sagamihara Hospital Research Ethics Committee, Kumamoto Center for Arthritis and Rheumatology Research Ethics Committee, Nagasaki Medical Center Research Ethics Committee, Yokohama Minami Kyosai Hospital Research Ethics Committee, and University of Tsukuba Research Ethics Committee. Written informed consent was obtained from all study participants. This study was conducted in accordance with the principles expressed in the Declaration of Helsinki.

RA patients were or were not diagnosed with ILD, based on the findings of chest radiography or computed tomography (CT) images. Images were reviewed by two physicians specializing in CVD-ILD, and categorized from A to Z, according to the Sagamihara Criteria as follows, A: Findings consistent with ILD, including usual interstitial pneumonia (honeycombing), non-specific interstitial pneumonia, and ground-glass attenuation patterns, were observed in high resolution CT (HRCT) images (length of shorter diameter of the lesion was ≥2 cm in a transverse section) [14], B: In case HRCT images were unavailable, evidence of ILD was found in conventional chest CT images (length of shorter diameter of the lesion was ≥2 cm in a transverse section); C: Evidence of ILD was observed in HRCT images (length of shorter diameter of the lesion was <2 cm in any transverse section); D: If HRCT images were unavailable, evidence of ILD was observed in conventional chest CT images (length of shorter diameter of the lesion was <2 cm in any transverse section); E: In case CT images were unavailable, evidence of ILD was observed in chest radiograms; F: In case CT images were unavailable, abnormalities were not observed in chest radiograms; G: If HRCT images were unavailable, abnormalities were not observed in conventional chest CT images; H: HRCT images were normal; X: Findings from lung HRCT images were predominantly other than ILD, including bronchiectasis, bronchiolitis, emphysema, organizing pneumonia, tuberculosis, and cancer; Y: If HRCT images were unavailable, findings from conventional chest CT images were predominantly other than ILD; Z: If CT images were unavailable, findings from chest radiograms were predominantly other than ILD. In this study, RA cases in categories A to D were diagnosed to be “ILD positive RA group” and those in G and H were diagnosed to be “ILD negative RA”. RA cases with other collagen diseases or in categories E, F, X, Y, or Z were excluded from this study. RA patients who visited Sagamihara Hospital (n = 2316) were classified as ILD positive RA (A to D; n = 247, 10.7%) and ILD negative RA (G, H; n = 431), according to the available CT images (n = 1034).

Rheumatoid Factor and anti-citrullinated peptide antibody were detected using N-latex RF kit (Siemens Healthcare Diagnostics, München, Germany) and Mesacup-2 test CCP (Medical & Biological Laboratories, Nagoya, Japan), respectively.

**Table 2:** HLA class II allele frequency in the RA patients.

	ILD(+)RA	ILD(−)RA	*P*	OR	*Pc*	95%CI
DRB1*01	21 (8.1)	44 (6.8)	0.5692	1.20	NS	
DRB1*04	71 (27.5)	257 (40.1)	0.0004	0.57	0.005	(0.41−0.78)
DRB1*07	0 (0)	2 (0.3)	1	0	NS	
DRB1*08	11 (4.2)	38 (5.9)	0.4167	0.71	NS	
DRB1*09	43 (16.6)	103 (16.0)	0.8419	1.04	NS	
DRB1*10	2 (0.7)	5 (0.7)	1	0.99	NS	
DRB1*11	3 (1.1)	8 (1.2)	1	0.93	NS	
DRB1*12	13 (5.0)	30 (4.6)	0.8632	1.08	NS	
DRB1*13	15 (5.8)	25 (3.9)	0.2141	1.52	NS	
DRB1*14	18 (6.9)	32 (5.0)	0.2610	1.43	NS	
DRB1*15	55 (21.3)	95 (14.8)	0.0228	1.55	0.296	(1.07−2.25)
DRB1*16	6 (2.3)	1 (0.1)	0.0029	15.2	0.037	(1.82−127.01)
DQB1*02	0 (0)	2 (0.3)	1	0	NS	
DQB1*03	73 (28.5)	182 (28.3)	1	1.01	NS	
DQB1*04	63 (24.6)	233 (36.2)	0.0007	0.57	0.004	(0.41−0.79)
DQB1*05	38 (14.8)	77 (11.9)	0.2688	1.28	NS	
DQB1*06	82 (32.0)	148 (23.0)	0.0067	1.57	0.033	(1.14−2.17)
DPB1*02	70 (27.1)	206 (32.0)	0.1509	0.79	NS	
DPB1*03	7 (2.7)	28 (4.3)	0.3400	0.61	NS	
DPB1*04	42 (16.2)	103 (16.0)	0.9204	1.02	NS	
DPB1*05	96 (37.2)	227 (35.3)	0.6448	1.08	NS	
DPB1*06	1 (0.3)	5 (0.7)	0.6797	0.50	NS	
DPB1*09	25 (9.6)	47 (7.3)	0.2766	1.36	NS	
DPB1*13	5 (1.9)	7 (1.0)	0.3403	1.79	NS	
DPB1*14	8 (3.1)	10 (1.5)	0.1846	2.02	NS	
DPB1*17	0 (0)	1 (0.1)	1	0	NS	
DPB1*19	2 (0.7)	3 (0.4)	0.6282	1.66	NS	
DPB1*38	0 (0)	2 (0.3)	1	0	NS	
DPB1*41	1 (0.3)	2 (0.3)	1	1.25	NS	
DPB1*47	1 (0.3)	1 (0.1)	0.4914	2.49	NS	

ILD(+)RA: ILD positive RA, ILD(−)RA: ILD negative RA, OR: odds ratio, 95%CI: confidence interval, *Pc*: corrected *P* value, NS: not significant. Allele frequencies are shown in parenthesis (%). Association was tested by Fisher’s exact test using 2×2 contingency tables.

**Table 3:** HLA-DRB1 allele frequency in the RA patients.

	ILD(+)RA	ILD(−)RA	*P*	OR	*Pc*	95%CI
DRB1*01∶01	21 (8.1)	44 (6.9)	0.5692	1.20	NS	
DRB1*04∶01	6 (2.3)	28 (4.4)	0.1775	0.52	NS	
DRB1*04∶03	4 (1.6)	7 (1.1)	0.5224	1.42	NS	
DRB1*04∶04	0 (0)	1 (0.2)	1	0	NS	
DRB1*04∶05	56 (21.7)	194 (30.3)	0.0106	0.64	0.2872	(0.45−0.90)
DRB1*04∶06	2 (0.8)	10 (1.6)	0.5251	0.49	NS	
DRB1*04∶07	0 (0)	1 (0.2)	1	0	NS	
DRB1*04∶10	3 (1.2)	16 (2.5)	0.3055	0.46	NS	
DRB1*07∶01	0 (0)	2 (0.3)	1	0	NS	
DRB1*08∶02	1 (0.4)	12 (1.9)	0.1239	0.20	NS	
DRB1*08∶03	10 (3.9)	25 (3.9)	1	0.99	NS	
DRB1*08∶23	0 (0)	1 (0.2)	1	0	NS	
DRB1*09∶01	43 (16.7)	103 (16.1)	0.8419	1.04	NS	
DRB1*10∶01	2 (0.8)	5 (0.8)	1	0.99	NS	
DRB1*11∶01	3 (1.2)	8 (1.3)	1	0.93	NS	
DRB1*12∶01	7 (2.7)	21 (3.3)	0.8324	0.82	NS	
DRB1*12∶02	6 (2.3)	9 (1.4)	0.3881	1.67	NS	
DRB1*13∶02	15 (5.8)	25 (3.9)	0.2141	1.52	NS	
DRB1*14∶02	1 (0.4)	0 (0)	0.2873	7.46	NS	
DRB1*14∶03	4 (1.6)	3 (0.5)	0.1093	3.34	NS	
DRB1*14∶05	5 (1.9)	2 (0.3)	0.0234	6.30	0.6315	(1.22−32.70)
DRB1*14∶06	3 (1.2)	6 (0.9)	0.7215	1.24	NS	
DRB1*14∶07	0 (0)	1 (0.2)	1	0	NS	
DRB1*14∶54	5 (1.9)	20 (3.1)	0.3794	0.61	NS	
DRB1*15∶01	23 (8.9)	39 (6.1)	0.1459	1.51	NS	
DRB1*15∶02	32 (12.4)	56 (8.8)	0.1068	1.48	NS	
DRB1*16∶02	6 (2.3)	1 (0.2)	0.0029	15.21	0.0773	(1.82−127.01)
DQB1*02∶01	0 (0)	2 (0.3)	1	0	NS	
DQB1*03∶01	24 (9.4)	59 (9.2)	0.8993	1.02	NS	
DQB1*03∶02	5 (2.0)	33 (5.1)	0.0415	0.37	0.5813	(0.14−0.95)
DQB1*03∶03	43 (16.8)	89 (13.9)	0.2964	1.25	NS	
DQB1*03∶06	1 (0.4)	1 (0.2)	0.4891	2.51	NS	
DQB1*04∶01	60 (23.4)	215 (33.5)	0.0030	0.61	0.0423	(0.44−0.85)
DQB1*04∶02	3 (1.2)	18 (2.8)	0.2195	0.41	NS	
DQB1*05∶01	24 (9.4)	52 (8.1)	0.5953	1.17	NS	
DQB1*05∶02	6 (2.3)	16 (2.5)	1	0.94	NS	
DQB1*05∶03	8 (3.1)	9 (1.4)	0.1039	2.27	NS	
DQB1*06∶01	42 (16.4)	86 (13.4)	0.2463	1.27	NS	
DQB1*06∶02	25 (9.8)	38 (5.9)	0.0587	1.72	0.8224	
DQB1*06∶04	14 (5.5)	24 (3.7)	0.2709	1.49	NS	
DQB1*06∶09	1 (0.4)	0 (0)	0.2851	0	NS	

ILD(+)RA: ILD positive RA, ILD(−)RA: ILD negative RA, OR: odds ratio, 95%CI: confidence interval, *Pc*: corrected *P* value, NS: not significant. Allele frequencies are shown in parenthesis (%). Association was tested by Fisher’s exact test using 2×2 contingency tables.

### Genotyping

Genotyping of *HLA-DRB1, DQB1*, and *DPB1* was performed by polymerase chain reaction using sequence-specific oligonucleotide probes, WAKFlow HLA typing kits (Wakunaga, Hiroshima, Japan) using Bio-Plex 200 system (Bio-Rad, Hercules, CA), or by MPH-2 HLA typing kits (Wakunaga). *HLA-DRB1* alleles encoding the SE were as follows: *01∶01, *04∶01, *04∶04, *04∶05, *04∶10, *10∶01, *14∶02, and *14∶06 [4]. *HLA-DRB1* locus of one RA patient without ILD and *HLA-DQB1* locus of one RA patient with ILD failed to be typed. We then attempted to type these two loci by Allele SEQR HLA-DRB1 and HLA-DQ sequence-based typing kit (Abbott Laboratories, Abbott Park, IL) and the data were analyzed by Assign SBT v3.5 (Conexio Genomics, Perth, Australia). Again, typing of these two loci failed, suggesting the possibility of novel alleles. Previously reported results of *HLA-DRB1* genotyping for 265 healthy controls were combined with our genotyping results and used for association analyses in this study [12]. The phase of *DRB1-DQB1* haplotype for each subject was inferred by PHASE v2.1 [15], [16].

### Statistical Analysis

Differences of RA characteristics or allele frequencies were analyzed by Student’s t-test, chi-square analysis or Fisher’s exact test using 2×2 contingency tables. Adjustment for multiple comparisons was performed using the Bonferroni method. Corrected *P* (*Pc*) value was calculated by multiplying the *P* value by the number of alleles tested. The sample size of this study (165 ILD positive RA patients and 432 ILD negative RA patients) had the power of 80% to detect association when the genotype relative risk was 0.71 or lower (SE) and 1.48 or higher (DR2), respectively [17].

For the meta-analysis of *HLA-DRB1*, our data and previously published data without overlap [11] were pooled using the DerSimonian-Laird method with the random effects model [18]. Lack of heterogeneity between the 2 populations was examined using the Breslow-Day test of homogeneity.

To examine whether *HLA-DRB1* and characteristics of RA independently contributes to susceptibility to ILD in RA, multiple logistic regression analysis was employed. Dominant, codominant and recessive models were tested for SE or DR2, and the model that provided the lowest *P* value was selected as the best fit model. As a result, the following were used as independent variables: SE, SE(−)/SE(−) = 0, SE(+)/SE(−) = 0 and SE(+)/SE(+) = 1 under the recessive model for SE; DR2, DR2(−)/DR2(−) = 0, DR2(+)/DR2(−) = 1, DR2(+)/DR2(+) = 1 under the dominant model for DR2, respectively. The deviation from 0 was evaluated for partial regression coefficients by the Wald test.

**Table 4:** HLA-DRB1 allele frequency in the RA patients and controls.

	ILD(+)RA	ILD(−)RA	Control	(ILD(+)RA vs Control)			95%CI	(ILD(−)RA vs Control)			95%CI
				*P*	OR	*Pc*		*P*	OR	*Pc*	
DRB1*01	21 (8.1)	44 (6.9)	29 (4.5)	0.0364	1.88	0.4370	(1.05−3.36)	0.0712	1.57	0.8547	
DRB1*04	71 (27.5)	257 (40.2)	150 (23.3)	0.1989	1.25	NS		8.1×10^−11^	2.21	9.7×10^−10^	(1.74−2.81)
DRB1*07	0 (0)	2 (0.3)	2 (0.3)	1	0	NS		1	1.01	NS	
DRB1*08	11 (4.3)	38 (5.9)	70 (10.9)	0.0012	0.37	0.014	(0.19−0.70)	0.0017	0.52	0.0207	(0.34−0.78)
DRB1*09	43 (16.7)	103 (16.1)	105 (16.3)	0.9208	1.03	NS		0.9397	0.98	NS	
DRB1*10	2 (0.8)	5 (0.8)	2 (0.3)	0.3236	2.51	NS		0.2864	2.53	NS	
DRB1*11	3 (1.2)	8 (1.3)	7 (1.1)	1	1.07	NS		0.8019	1.15	NS	
DRB1*12	13 (5.0)	30 (4.7)	35 (5.4)	0.8711	0.92	NS		0.6110	0.86	NS	
DRB1*13	15 (5.8)	25 (3.9)	68 (10.6)	0.0296	0.52	0.356	(0.29−0.93)	4.4×10^−6^	0.34	5.2×10^−5^	(0.21−0.55)
DRB1*14	18 (7.0)	32 (5.0)	61 (9.5)	0.2967	0.72	NS		0.0024	0.50	0.0291	(0.32−0.78)
DRB1*15	55 (21.3)	95 (14.8)	107 (16.6)	0.1031	1.36	NS		0.3998	0.87	NS	
DRB1*16	6 (2.3)	1 (0.2)	8 (1.2)	0.2416	1.89	NS		0.0384	0.12	0.4610	(0.02−1.00)
SE(+)	92 (35.7)	294 (45.9)	145 (22.5)	0.0001	1.91		(1.39−2.61)	9.0×10^−19^	2.92		(2.30−3.72)

ILD(+)RA: ILD positive RA, ILD(−)RA: ILD negative RA, OR: odds ratio, 95%CI: confidence interval, *Pc*: corrected *P* value, NS: not significant. Allele frequencies are shown in parenthesis (%). Association was tested by Fisher’s exact test using 2×2 contingency tables.

## Results

### Characteristics of ILD Positive RA and ILD Negative RA Groups

Characteristics of ILD positive RA and ILD negative RA groups were described in Table 1. Mean age, percentage of males, disease duration, and positivity of rheumatoid factor in ILD positive RA group were higher than that of ILD negative RA group. The difference of smoking habit, anti-citrullinated peptide antibody, or Steinbrocker stage was not detected.

### Association of HLA-DR and DQ with ILD

We tested whether *HLA class II* was associated with ILD in the comparison of ILD positive RA and ILD negative RA groups. Low-resolution and high-resolution typing data are shown in Table 2 and 3, respectively. A significant association was found for DRB1*04 alleles with resistance to ILD (*Pc* = 0.0054, odds ratio [OR] 0.57, 95% confidence interval [CI] 0.41−0.78, Table 2). On the other hand, DRB1*16 allele (DRB1*16∶02 in the high-resolution typing) was associated with susceptibility to ILD (*Pc* = 0.0372, OR 15.21, 95%CI 1.82−127.01, Table 2 and 3). HLA-DQB1*04 allele was also associated with resistance to ILD (*Pc* = 0.0036, OR 0.57, 95%CI 0.41−0.79). DQB1*06 allele was associated with susceptibility to ILD (*Pc* = 0.0333, OR 1.57, 95%CI 1.14−2.17). Thus, there was an association of *HLA-DR* and *DQ* with the susceptibility or resistance to ILD in RA patients.

We then compared the allele frequency of *HLA-DR* in ILD positive RA group with healthy controls (Table 4). Although DRB1*04, *08, *13, and *14 were associated with ILD negative RA, association of DRB1*04 with ILD positive RA was not observed.

The *DRB1* and *DQB1* alleles which showed significant association are in strong linkage disequilibrium. In order to elucidate which of the *DRB1* and *DQB1* was responsible for the association, haplotype analysis of *DRB1-DQB1* was performed. Influence of the presence or absence of a haplotype on ILD phenotype was analyzed by Fisher’s exact test using 2×2 contingency tables under the dominant model. DRB1*16∶02-DQB1*05∶02 was significantly associated with ILD positive RA (n = 4 [1.6%] in ILD positive RA vs. n = 1 [0.2%] in ILD negative RA, *P* = 0.0257, OR 10.09), but DRB1*14∶54-DQB1*05∶02 was not (n = 2 [0.8%] in ILD positive RA vs. n = 13 [2%] in ILD negative RA, *P* = 0.2544, OR 0.38). The two-locus analysis was also performed to clarify the primary role of DRB1*16∶02 or DQB1*05∶02. OR for DRB1*16∶02 in the patients without DQB1*05∶02 was 12.61, while OR for DQB1*05∶02 in the patients without DRB1*16∶02 was 0.36 (Table 5). The difference did not reach statistical significance, because the strong linkage disequilibrium between DRB1*16∶02 and DQB1*05∶02 causes the low frequency of DRB1*16∶02 in the patients without DQB1*05∶02 and that of DQB1*05∶02 in the patients without DRB1*16∶02. Thus, haplotype and two-locus analysis suggested the primary role of DRB1*16∶02.

### Association of SE and DR2 with ILD

The effect of SE on ILD in RA was evaluated. Frequency of SE alleles was lower in ILD positive RA group compared with ILD negative RA group (*P* = 0.0055, OR 0.66, 95%CI 0.49−0.88). This association was also observed in the genotype frequency of SE under the recessive model (*P* = 0.0012, OR 0.31, 95%CI 0.15−0.65). However, the association was not confirmed under the dominant model (*P* = 0.0968, OR 0.69, 95%CI 0.45−1.07), suggesting that having two copies of SE confers resistance to ILD development in RA. The effect of DR2 serological group, consisting of DRB1*15, and *16,* on ILD in RA was also evaluated. Frequency of DR2 alleles was higher in ILD positive RA compared with ILD negative RA group (*P* = 0.0020, OR 1.75, 95%CI 1.22−2.51). Association with DR2 was also observed under the dominant model (*P* = 0.0007, OR 2.06, 95%CI 1.35−3.16).

**Table 5:** *HLA-DRB1* or *DQB1* allele carrier frequency in the RA cases with specific *DQB1* or *DRB1* alleles (two locus analysis).

DRB1-DQB1	DQB1	ILD(+)RA		ILD(−)RA		*P*	OR	*Pc*	95%CI	DRB1	ILD(+)RA		ILD(−)RA		*P*	OR	*Pc*	95%CI
		DRB1 allele positivity		DRB1 allele positivity							DQB1 allele positivity		DQB1 allele positivity					
		(+)	(−)	(+)	(−)						(+)	(−)	(+)	(−)				
*15∶01−*06∶02	(+)	21 (95.5)	1 (4.5)	36 (100)	0 (0)	0.3793	0	NS		(+)	21 (95.5)	1 (4.5)	36 (97.3)	1 (2.7)	1	0.58	NS	
	(−)	1 (0.9)	106 (99.1)	1 (0.4)	284 (99.6)	0.4719	2.68	NS		(−)	1 (0.9)	106 (99.1)	0 (0)	284 (100)	0.2737	8.01	NS	
*15∶02−*06∶01	(+)	30 (78.9)	8 (21.1)	51 (68.0)	24 (32.0)	0.2726	1.76	NS		(+)	30 (96.8)	1 (3.2)	51 (100)	0 (0)	0.3780	0	NS	
	(−)	1 (1.1)	90 (98.9)	0 (0)	246 (100)	0.2700	8.17	NS		(−)	8 (8.2)	90 (91.8)	24 (8.9)	246 (91.1)	1	0.91	NS	
*16∶02−*05∶02	(+)	4 (66.7)	2 (33.3)	1 (6.7)	14 (93.3)	0.0114	28.00	0.0912	(1.99−394.40)	(+)	4 (66.7)	2 (33.3)	1 (100)	0 (0)	1	0	NS	
	(−)	2 (1.6)	121 (98.4)	0 (0)	306 (100)	0.0817	12.61	0.6536		(−)	2 (1.6)	121 (98.4)	14 (4.4)	306 (95.6)	0.2549	0.36	NS	
*15∶02−*05∶02	(+)	1 (16.7)	5 (83.3)	3 (20.0)	12 (80.0)	1	0.80	NS		(+)	1 (3.2)	30 (96.8)	3 (5.9)	48 (94.1)	1	0.53	NS	
	(−)	30 (24.4)	93 (75.6)	48 (15.7)	258 (84.3)	0.0385	1.73	0.3080	(1.04−2.90)	(−)	5 (5.1)	93 (94.9)	12 (4.4)	258 (95.6)	0.7820	1.16	NS	

ILD(+)RA: ILD positive RA, ILD(−)RA: ILD negative RA, OR: odds ratio, 95%CI: confidence interval, *Pc*: corrected *P* value, NS: not significant. Allele frequencies are shown in parenthesis (%). Association was tested by Fisher’s exact test using 2×2 contingency tables.

We then made an attempt to confirm these associations in an independent set of ILD positive RA and ILD negative RA. The frequency of SE alleles was lower in ILD positive RA group compared with ILD negative RA group (24 [33.3%] in ILD positive RA vs. 84 [38.2%] in ILD negative RA, *P* = 0.4595, OR 0.81, 95%CI 0.46−1.42), although the difference did not reach significance. On the other hand, significant increase of DR2 alleles in ILD positive RA was replicated (20 [27.8%] in ILD positive RA vs. 37 [16.8%] in ILD negative RA, *P* = 0.0417, OR 1.90, 95%CI 1.02−3.55). When these data were combined with that of RA patients from Sagamihara hospital, both the protective association of SE (*P* = 0.0064, OR 0.69, 95%CI 0.53−0.90, Table 6) and the positive association of DR2 group (*P* = 0.0002, OR 1.78, 95%CI 1.31−2.43, Table 6) remained significant.

Meta-analysis of these data combined with the data from the previous report [11] showed the protective association of SE (pooled *P* = 0.0069, OR 0.72, 95%CI 0.57−0.92) and the positive association of DR2 group (pooled *P* = 0.0001, OR 1.96, 95%CI 1.39−2.75). Breslow-Day test confirmed the lack of heterogeneity between our data and the previously reported data on SE and DR2 (*P* = 0.4848 and 0.2767, respectively).

We then examined whether the protective effect of SE is secondary to the increase of DR2 group in the ILD positive RA group, by excluding patients with DR2 from the analysis. SE was still associated with resistance to ILD (*P* = 0.0270, OR 0.69, 95%CI 0.49−0.96, Table 6), indicating that the protective association of SE was not caused by the positive association of DR2.

We examined whether the positive association of DR2 group is secondary to the decrease of SE in the ILD positive RA patients. When the patients with SE were excluded from the analysis, the DR2 allele frequency was still higher in ILD positive RA than ILD negative RA, although the effect was not statistically significant (*P* = 0.1108, OR 1.46, 95%CI 0.92−2.32, Table 6).

To examine the independent contribution of SE and characteristics of RA to susceptibility to ILD, multiple logistic regression analysis was conducted. The association of age (*P* = 2.2×10^−7^, OR 1.08, 95%CI 1.05−1.12), male gender (*P* = 0.0003, OR 3.60, 95%CI 1.81−7.18) and SE (*P* = 0.0091, OR 0.34 95%CI 0.15−0.76) with ILD was significant. On the other hand, significant association of smoking habit, disease duration, rheumatoid factor, anti-citrullinated peptide antibody, or Steinbrocker stage with ILD was not observed. Similar results were obtained from the multiple logistic regression analysis on DR2 and characteristics of RA. The association of age (*P* = 1.0×10^−7^, OR 1.09, 95%CI 1.05−1.12), male gender (*P* = 0.0005, OR 3.39, 95%CI 1.71−6.71) and DR2 (*P* = 0.0080, OR 2.02 95%CI 1.20−3.40) with ILD remained significant, though that of smoking habit, disease duration, rheumatoid factor, anti-citrullinated peptide antibody, or Steinbrocker stage with ILD was not observed.

## Discussion

Several studies have shown that *HLA-DR* contributes to the susceptibility to extra-articular manifestations of RA. The association of Felty’s syndrome, one of extra-articular manifestations of RA, with SE was reported [5]. However, few studies have focused on the association of *HLA* with ILD in RA, one of the potentially life-threatening extra-articular manifestations. To our knowledge, this is the first report of association of the SE with an obvious protective effect for ILD in RA, although a weak protective effect by SE for ILD in RA was previously observed [11], [19]. Our findings suggest different pathogenic mechanisms between ILD and Felty’s syndrome in RA.

**Table 6:** SE and DR2 frequency in the all RA patients and RA patients without SE or DR2.

	Allele frequency		Genotype frequency			Allelic association			Dominant model			Recessive model		
	SE(+)	SE(−)	SE(+)/SE(+)	SE(+)/SE(−)	SE(−)/SE(−)	*P*	OR	(95%CI)	*P*	OR	(95%CI)	*P*	OR	(95%CI)
All RA patients														
ILD(+)RA	116 (35.2)	214 (64.8)	11 (6.7)	94 (57.0)	60 (36.4)	0.0064	0.69	(0.53−0.90)	0.1172	0.74	(0.51−1.08)	0.0008	0.34	(0.18−0.66)
ILD(-)RA	378 (43.9)	484 (56.1)	75 (17.4)	228 (52.9)	128 (29.7)									
RA patients without DR2														
ILD(+)RA	77 (41.8)	107 (58.2)	11 (12.0)	55 (59.8)	26 (28.3)	0.0270	0.69	(0.49−0.96)	0.2257	0.72	(0.43−1.22)	0.0107	0.42	(0.21−0.83)
ILD(-)RA	314 (51.1)	300 (48.9)	75 (24.4)	164 (53.4)	68 (22.1)									
	**DR2(+)**	**DR2(−)**	**DR2(+)/DR2(+)**	**DR2(+)/DR2(−)**	**DR2(−)/DR2(−)**									
All RA patients														
ILD(+)RA	81 (24.5)	249 (75.5)	8 (4.8)	65 (39.4)	92 (55.8)	0.0002	1.78	(1.31−2.43)	0.0003	1.96	(1.36−2.85)	*0.0955	2.39	(0.91−6.30)
ILD(-)RA	133 (15.4)	729 (84.6)	9 (2.1)	115 (26.7)	307 (71.2)									
RA patients without SE														
ILD(+)RA	42 (35.0)	78 (65.0)	8 (13.3)	26 (43.3)	26 (43.3)	0.1108	1.46	(0.92−2.32)	0.2107	1.48	(0.80−2.75)	0.1602	2.03	(0.74−5.57)
ILD(-)RA	69 (27.0)	187 (73.0)	9 (7.0)	51 (39.8)	68 (53.1)									

ILD(+)RA: ILD positive RA, ILD(−)RA: ILD negative RA, OR: odds ratio, 95%CI: confidence interval, SE: Shared epitope. Genotype or allele frequencies are shown in parenthesis (%). Association was tested by chi-square analysis or Fisher’s exact test using 2×2 contingency tables under the indicated models for SE or DR2. *Fisher’s exact test was employed.

When SE frequency in ILD positive RA group was compared with healthy controls, the frequency of SE alleles was still significantly higher in ILD positive RA group (*P* = 0.0001, OR 1.91, 95%CI 1.39−2.61 under the allele model, Table 4, *P* = 1.7×10^−6^, OR 2.77, 95%CI 1.81−4.23 under the dominant model); however, the association was considerably weaker as compared with ILD negative RA (*P* = 9.0×10^−19^, OR 2.92, 95%CI 2.30−3.72 under the allele model, Table 4, *P* = 3.3×10^−17^, OR 4.05, 95%CI 2.90−5.64 under the dominant model). Although the implication of this finding is not clear, this might suggest that the role of SE may not be as important in ILD positive RA as compared with RA *per se*. Environmental triggers might play a substantial role in the development of ILD. Alternatively, the genetic background of ILD positive RA and ILD negative RA groups may be different, and genetic factors other than SE may play a significant role in the former. Significantly higher proportion of male patients in ILD positive RA might support this possibility. Such possibilities are not mutually exclusive, and some of them are currently being tested.

The association of DRB1*15∶02 and ILD in RA has been reported in the Japanese population [11]. Notably, DRB1*15∶01 and *16∶02 frequency was higher in idiopathic pulmonary fibrosis patients [20], [21]. DRB1*15, and 16 belong to the DR2 serological group. DR2 was reported to be the susceptibility allele of systemic lupus erythematosus [22], other autoimmune diseases, and other extra-articular manifestations in RA [23], [24]. The association of DR2 with ILD in RA was also confirmed in our study. A common peptide (DENPVVHFFKNIVTPRTPP) is known to be presented by DRB1*15∶01 and *16∶02*, explaining the susceptibility of DR2 positive individuals for ILD in RA [25].

Although DRB1*11 was reported to be associated with pulmonary fibrosis in European systemic sclerosis patients [26], this association was not confirmed in our study. This could be explained by the differences in the pathogenesis of ILD in RA and systemic sclerosis, ethnic difference, or both. At this point, we cannot rule out the possibility that another causative genes might exist in the *HLA* region in linkage disequilibrium with the culprit gene in the *DR* locus. This possibility could be addressed by re-sequencing the entire *HLA* region of DRB1*16∶02 allele.

The logistic regression analysis indicated that the association of age and gender with ILD in RA. These data suggested the existence of a subset of RA with older age at onset and higher ratio of male that is susceptible for ILD. Because of the limited sample size of this study, the observed statistical association was modest. The association of *HLA-DR* should be confirmed in future independent studies. Since the allelic distribution of *HLA* in other ethnic populations is different from that in the Japanese, the role of *HLA-DR* for ILD in RA in other populations should be determined.

This is the first identification of association of HLA-DR*16 with susceptibility to, and that of the SE with resistance to ILD in RA. Our findings support the role of *HLA-DR* in CVD-ILD pathogenesis.

## References

[1] Perricone C, Ceccarelli F, Valesini G (2011). An overview on the genetic of rheumatoid arthritis: a never-ending story..

[2] Scott IC, Steer S, Lewis CM, Cope AP (2011). Precipitating and perpetuating factors of rheumatoid arthritis immunopathology: linking the triad of genetic predisposition, environmental risk factors and autoimmunity to disease pathogenesis.. Best Pract Res Clin Rheumatol.

[3] Lewis SN, Nsoesie E, Weeks C, Qiao D, Zhang L (2011). Prediction of disease and phenotype associations from genome-wide association studies. PLoS ONE 6: e27175.. Epub 22011 Nov.

[4] Reveille JD (1998). The genetic contribution to the pathogenesis of rheumatoid arthritis.. Curr Opin Rheumatol.

[5] Turesson C, Weyand CM, Matteson EL (2004). Genetics of rheumatoid arthritis: Is there a pattern predicting extraarticular manifestations?. Arthritis Rheum.

[6] Lanchbury JS, Jaeger EE, Sansom DM, Hall MA, Wordsworth P (1991). Strong primary selection for the Dw4 subtype of DR4 accounts for the HLA-DQw7 association with Felty’s syndrome.. Hum Immunol.

[7] Kim HY, Min JK, Yang HI, Park SH, Hong YS (1997). The impact of HLA-DRB1*0405 on disease severity in Korean patients with seropositive rheumatoid arthritis.. Br J Rheumatol.

[8] Weyand CM, Xie C, Goronzy JJ (1992). Homozygosity for the HLA-DRB1 allele selects for extraarticular manifestations in rheumatoid arthritis.. J Clin Invest.

[9] Turesson C, Jacobsson LT (2004). Epidemiology of extra-articular manifestations in rheumatoid arthritis.. Scand J Rheumatol.

[10] Koduri G, Norton S, Young A, Cox N, Davies P (2010). Interstitial lung disease has a poor prognosis in rheumatoid arthritis: results from an inception cohort.. Rheumatology.

[11] Migita K, Nakamura T, Koga T, Eguchi K (2010). HLA-DRB1 alleles and rheumatoid arthritis-related pulmonary fibrosis.. J Rheumatol.

[12] Shibue T, Tsuchiya N, Komata T, Matsushita M, Shiota M (2000). Tumor necrosis factor alpha 5′-flanking region, tumor necrosis factor receptor II, and HLA-DRB1 polymorphisms in Japanese patients with rheumatoid arthritis.. Arthritis Rheum.

[13] Arnett FC, Edworthy SM, Bloch DA, McShane DJ, Fries JF (1988). The American Rheumatism Association 1987 revised criteria for the classification of rheumatoid arthritis.. Arthritis Rheum.

[14] Webb WR (2006). Thin-section CT of the secondary pulmonary lobule: anatomy and the image–the 2004 Fleischner lecture.. Radiology.

[15] Stephens M, Donnelly P (2003). A comparison of bayesian methods for haplotype reconstruction from population genotype data. Am J Hum Genet 73: 1162–1169.. Epub 2003 Oct.

[16] Stephens M, Smith NJ, Donnelly P (2001). A new statistical method for haplotype reconstruction from population data.. Am J Hum Genet.

[17] Ohashi J, Yamamoto S, Tsuchiya N, Hatta Y, Komata T (2001). Comparison of statistical power between 2 * 2 allele frequency and allele positivity tables in case-control studies of complex disease genes.. Ann Hum Genet.

[18] DerSimonian R, Laird N (1986). Meta-analysis in clinical trials.. Control Clin Trials.

[19] Turesson C, Schaid DJ, Weyand CM, Jacobsson LT, Goronzy JJ (2005). The impact of HLA-DRB1 genes on extra-articular disease manifestations in rheumatoid arthritis.. Arthritis Res Ther.

[20] Xue J, Gochuico BR, Alawad AS, Feghali-Bostwick CA, Noth I (2011). The HLA class II Allele DRB1*1501 is over-represented in patients with idiopathic pulmonary fibrosis.. PLoS ONE.

[21] Falfan-Valencia R, Camarena A, Juarez A, Becerril C, Montano M (2005). Major histocompatibility complex and alveolar epithelial apoptosis in idiopathic pulmonary fibrosis.. Hum Genet.

[22] Sirikong M, Tsuchiya N, Chandanayingyong D, Bejrachandra S, Suthipinittharm P (2002). Association of HLA-DRB1*1502-DQB1*0501 haplotype with susceptibility to systemic lupus erythematosus in Thais.. Tissue Antigens.

[23] Mattey DL, Gonzalez-Gay MA, Hajeer AH, Dababneh A, Thomson W (2000). Association between HLA-DRB1*15 and secondary Sjogren’s syndrome in patients with rheumatoid arthritis.. J Rheumatol.

[24] Tokunaga NK, Noda R, Kaneoka H, Ogahara S, Murata T (1999). Association between HLA-DRB1*15 and Japanese patients with rheumatoid arthritis complicated by renal involvement.. Nephron.

[25] Wucherpfennig KW, Sette A, Southwood S, Oseroff C, Matsui M (1994). Structural requirements for binding of an immunodominant myelin basic protein peptide to DR2 isotypes and for its recognition by human T cell clones.. J Exp Med.

[26] Simeon CP, Fonollosa V, Tolosa C, Palou E, Selva A (2009). Association of HLA class II genes with systemic sclerosis in Spanish patients.. J Rheumatol.

